# Comparison of Macular Thickness in Patients with Keratoconus and Control Subjects Using the Cirrus HD-OCT 

**DOI:** 10.1155/2015/832863

**Published:** 2015-06-08

**Authors:** R. L. Brautaset, R. Rosén, A. Cerviño, W. L. Miller, J. Bergmanson, M. Nilsson

**Affiliations:** ^1^Unit of Optometry, Department of Clinical Neuroscience, Karolinska Institutet, P.O. Box 8056, 10420 Stockholm, Sweden; ^2^Optics Department, University of Valencia, C/Dr. Moliner 50, 46100 Burjassot, Spain; ^3^TERTC, University of Houston, College of Optometry, Houston, TX 77204-2020, USA

## Abstract

*Purpose.* The aim of the present study was to compare macular thickness in patients with keratoconus (KC) with macular thickness in healthy subjects. *Subjects and Methods.* Twenty-six patients with KC and 52 control subjects were included. The macular structure was evaluated using a Zeiss Cirrus HD-OCT. The scan pattern used was 512 × 128, which covers an area of approximately 6 × 6 mm of the retina. The cube volume was assessed as well as macular thickness in each of the 9 sectors defined by the software. *Results.* The mean signal strength was significantly lower in the KC group (mean 8.4, range 6–10) compared with the control group (mean 9.7, range 7–10), *P* < 0.0001 (unpaired *t*-test). There were no significant differences in cube volume (unpaired *t*-test), cube average thickness, or macular thickness between the KC group and the control subjects in any of the retinal locations (one-way ANOVA). *Conclusion.* Macular structure as measured by OCT in KC subjects should be expected to lie within the range of age and sex matched controls.

## 1. Introduction

Keratoconus (KC) is a degenerative corneal disease, but the exact pathophysiologic process of keratoconus is still unknown. The abnormalities in keratoconus include the degeneration of epithelial basal cells and breaks accompanied by down growth of epithelium into the anterior limiting lamina, as well as the release of catabolic enzymes and biochemical cytokines that cause thinning of collagen matrix lamella, loss of corneal stroma, and apoptosis [1–3].

The morphological alterations of keratoconic corneas are usually observed at the apex of the cone but the peripheral cornea has also been shown to exhibit disease related changes [4, 5].

Keratoconus has been associated with other systemic and ocular conditions, for example, Ehlers-Danlos syndrome and Leber's congenital amaurosis [6]. Both of these conditions are able to cause retinal degeneration. There are also a few case reports, describing central serous chorioretinopathy (CSC) in patients with KC [7] and choroidal neovascularisation [8]. Even if it is unclear whether these conditions have any common pathological features, data all together suggests that posterior segment disorders might be associated with, or at least can coexist with, keratoconus. Therefore, a thorough retinal examination before corneal transplantation seems relevant to avoid exaggerated expectations on surgery outcome.

It is often difficult to visualize the fundus in patients with keratoconus because of high refractive errors and advanced corneal astigmatism as well as corneal opacities caused by striae and corneal scarring [9]. The limited view makes it sometimes hard for clinicians to reveal maculopathy preoperatively and there are occasions when patients have been diagnosed with maculopathy first after corneal transplantation. Although a clear graft has been implemented, best corrected visual acuity (BCVA) has not been satisfyingly improved in these cases [10].

For evaluation of macular structure* in vivo*, Optical Coherence Tomography (OCT) is nowadays a well-established method [11]. The technique has the potential to reveal macular lesions due to various conditions and it is valuable for visualization of, for example, macular oedema. OCT has been shown to be able to measure retinal thickness despite the reduced optical quality of the cornea in KC subjects [12–14]; however, very little data is available to demonstrate if macular thickness is normal or unaffected by the disease. Therefore, the aim of this study was to compare the macular thickness in patients with KC with macular thickness in healthy subjects.

## 2. Subjects and Methods

Twenty-six patients, with keratoconus and 52 control subjects, were recruited from the Texas Eye Research and Technology Centre (TERTC), at the University of Houston. To be included, a KC subject must manifest one or more of the following clinical signs: posterior stress lines (Vogt striae), Fleischer ring, external sign (Munson sign) together with a topography positive for KC (central corneal power superior to 48.7 D), and an inferior superior asymmetry above 1.9 [15–17]. Exclusion criteria included the following: any previous ocular surgery, the use of any systemic or ocular medications, and any chronic disorder that can affect the eye. Control subjects needed to fulfill the following criteria: asymptomatic, no ocular pathology, no history of ocular treatment, no medication with known effect on visual acuity, and visual acuity of 0.1 (Log MAR) or better. All participants in the KC group were patients from the clinic, while the control subjects were staff and students from the TERTC clinic as well as friends of the KC patients. For demographic data, see Table 1. For the KC group both eyes were included in the analysis if both eyes were affected by KC and in the control group only the right eye was included.

This study followed the tenants of the Declaration of Helsinki, was in accord with the Health Insurance Portability and Accountability Act of 1996, and was approved by the Committee for the Protection of Human Subjects of the University of Houston. Written informed consent was obtained from all KC patients and control subjects.

### 2.1. Observational Procedure

All patients and subjects underwent refraction to determine BCVA. They also underwent Orbscan measurements for determination of corneal astigmatism; see Table 1. Biomicroscopy was performed using a Haag Streit (BQ900) biomicroscope and all subjects were graded/classified by the same investigator (RB) and presence of prominent nerve fibres, Fleischer's ring, Vogt's striae, Munson's sign, and anterior/posterior corneal scarring was noted; see Table 2.

The severity of keratoconus was graded according to the CLEK system using the greatest corneal curvature based on Orbscan measurements [18]. Twenty-four of the patients' eyes were classified with grade 3, eighteen with grade 2, and six with grade 1.

### 2.2. Macular Structure Measurements

**Figure 1:**
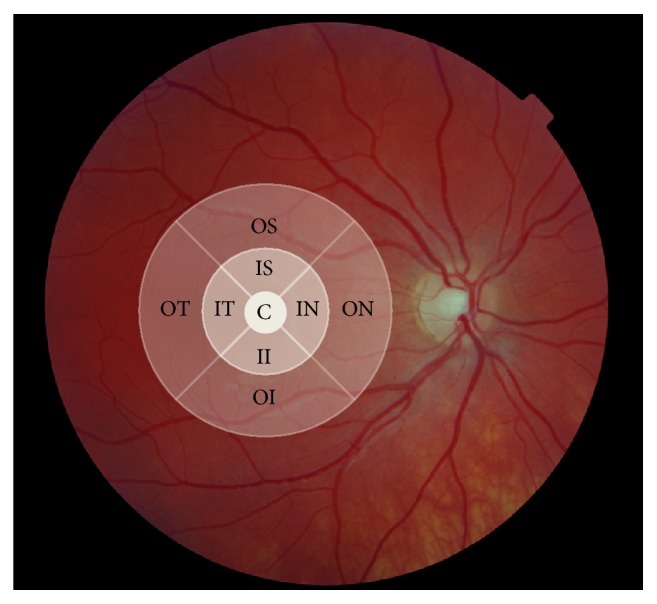
The cube volume and macular thickness was determined according to the 9 sectors originating from the Early Diabetic Retinopathy Study Group (C: centre, IS: inner superior, IN: inner nasal, II: inner inferior, IT: inner temporal, OS: outer superior, ON: outer nasal, OI: outer inferior, and OT: outer temporal).

The macular structure was evaluated using a Zeiss Cirrus HD-OCT. The scan pattern used was 512 × 128, which covers an area of approximately 6 × 6 mm of the retina. The cube volume was assessed as well as macular thickness in each of the 9 sectors defined by the software (originally from the Early Diabetic Retinopathy Study Group); see Figure 1. When needed, several scans were obtained and the scan with the highest quality/signal strength was used for analysis.

## 3. Results

**Figure 2:**
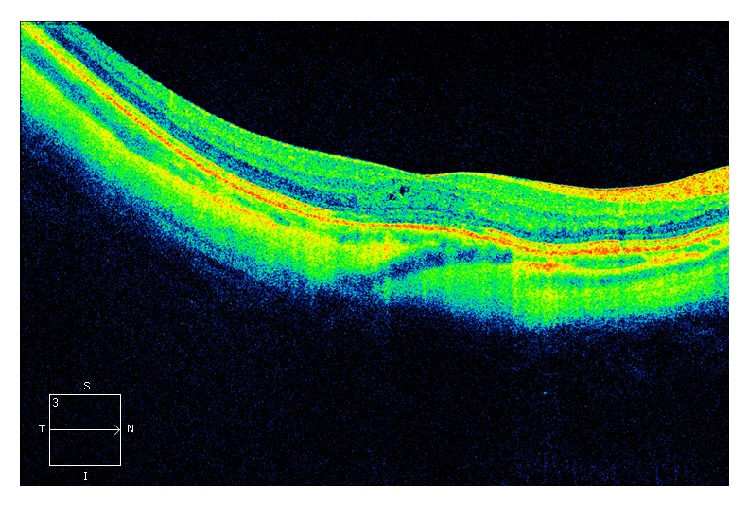
HD 5-line raster scan from one of the keratoconus patient showing signs of scarring after a choroidal lesion.

OCT scans were successfully obtained from all subjects except in one KC patient. Because of high refractive error, outside the limits of the machine (−30 D) the image quality was poor (signal strength 2) and the images were excluded from the analysis. Another patient in the KC group showed abnormal macular measurements with scarring after choroidal lesions in one eye. Although the image quality was very good (see Figure 2) and the measurements could be regarded as reliable, it did not seem appropriate for calculation of reference values and the data was excluded from further analysis.

The mean signal strength was significantly lower in the KC group (mean 8.4 ± 1.4 SD, range 6–10) compared with the control group (mean 9.7 ± 0.49 SD, range 7–10), *P* < 0.0001 (unpaired *t*-test).

There was a statistical significant correlation between signal strength and astigmatism (Sim's *K* value) (*P* > 0.001, *r* = −0.63) and between signal strength and visual acuity (*P* = 0.01, *r* = −0.37) in the KC group (Spearman rank correlation).

There were no significant differences (see Table 3) in cube volume (unpaired *t*-test), cube average thickness, or macular thickness between the KC group and the control subjects in any of the retinal locations (one-way ANOVA).

## 4. Discussion

Macular thickness measurements were successfully obtained from all KC patients except in one patient who had a high refractive error (>−30 D), outside the correction limits of the OCT machine used in this study (Zeiss Cirrus HD-OCT). On average, no difference in macular thickness could be found when comparing the KC group and controls. This finding is in line with the findings of Moschos et al. [13]. One KC subject had maculopathy which was easily detected and not masked although the patient had high refractive error (−24 D), prominent nerve fibres, Vogt's striae, and Fleisher's ring, indicating severe keratoconus or grade 3 according to the CLEK grading system [18]; see Figure 2. It could be argued if the maculopathy found in one of the KC subjects could be related to keratoconus, but that is outside the scope of this study. To answer such a question large cross-sectional studies are needed including not only OCT measurements. However, it is important information that macular changes seem easy to detect also in patients with poor optics caused by changes seen in KC. In 1996, Moschos and coworkers [19], performed electroretinogram (ERGs) and visual evoked potentials (VERs) in patients with keratoconus. In a handful of patients (5 out of 255) abnormal results were obtained and explained by coexisting diffuse or central tapetoretinal degeneration. The low visual acuity among those patients was, therefore, not only explained by the corneal lesions but also explained by the photoreceptor dysfunction. After successful corneal graft surgery, visual acuity was not increased and the transplantation of cornea in these cases was in vain. Preoperatively OCT examinations might have given useful information in these cases, which has been described in the case report by Meyer et al. [20].

In our subjects the signal strength of the OCT measurements were significantly lower in the KC group compared with the control subjects but all the images were possible to interpret. The signal strength ranged from 6 to 10, values which have been referred to as moderate to excellent [21]. Differences in signal strength have been proven to influence measurements but the differences have not been regarded as of any clinical importance [21]. In a recent study aiming to evaluate the effects of different parameters like signal strength, age, sex, and axial length on macular measurements using OCT, no parameter except age influenced the measurements [22]. Further, the influence of astigmatism on macular thickness measurements was recently evaluated by Hwang et al. [23]. They measured macular thickness in healthy subjects before and after fitting a soft contact lens inducing approximately 3 diopters astigmatism in 90 and 180 degrees, respectively. No changes in macular thickness could be found although retinal nerve fibre layer (RNFL) thickness measurements were affected and the range in mean difference with and without the lens was 0.75–5.11 *μ*m. Such small differences are, however, only clinically relevant when one, through follow up, is trying to detect degenerative changes and not when trying to detect an abnormal RNFL thickness. Cankaya and coworkers [24] recently described the outcome of RNFL thickness and optic nerve head (ONH) measurements in patients with KC. They found that RNFL thickness measurements were more comparable than ONH parameters when comparing patients with keratoconus and healthy subjects. Altogether, these two studies indicate that it is fully possible to obtain reliable data from measurements of retinal structures also in patients with keratoconus.

Since the values of the parameters analysed by the OCT in this study did not differ between KC subjects and controls, combined with the fact that several of the KC patients had pronounced signs of Keratoconus, abnormal OCT values from examination of patients with KC should be considered with gravity. Furthermore, the results from this study indicate that OCT should be regarded as a valuable instrument for macular evaluation in patients before corneal transplantation and used in order to improve the anticipation of the outcome of a corneal graft implant.

## 5. Conclusion

Even though the optical quality in aspects of astigmatism will influence the quality of OCT measurements, the current study indicates that macular structure as measured by OCT in KC subjects should be expected to lie within the range of age and sex matched controls.

## Figures and Tables

**Table 1:** Demographic data over study population.

	KC patients	Controls
Number of subjects	26	52
Number of eyes	48	52
Age (mean ± SD)	37.3 ± 12.2	37.6 ± 12.6
Male/female	13/13	25/27
Spherical equivalent (dioptres)	−9.0 (±7.6)	−2.5 (±3.5)
Visual acuity (log MAR)	0.35 (±0.3)	0.02 (±0.1)
Sim's *K* value		
Max. (dioptres)	52.30 ± 6.28	44.14 ± 1.40
Min. (dioptres)	57.29 ± 5.20	43.23 ± 1.42

Age, spherical equivalent, and visual acuity are given as mean values and standard deviations.

**Table 2:** Slit lamp findings.

Prominent nerve fibres	Fleischer's ring	Vogt's striae	Munson's sign	Anterior corneal scarring	posterior corneal scarring
38	15	47	3	9	21

Number of eyes with each clinical finding in the KC group.

**Table 3:** 

	Cube vol.	Cube average	Central	IS	II	IT	IN	OS	OI	OT	ON
KC eyes *n* = 44	9.9 ± 0.5	274.8 ± 13.9	254.1 ± 26.6	318.8 ± 17.9	316.8 ± 17.6	308.8 ± 21.1	317.2 ± 19.7	276.2 ± 16.6	270.6 ± 22.2	260.6 ± 18.7	280.7 ± 25.9

Control eyes *n* = 80	10.0 ± 0.5	276.8 ± 13.8	257.8 ± 15.9	32.0 ± 13.7	320.8 ± 14.4	312.2 ± 14.3	322.2 ± 18.0	278.0 ± 11.7	275.3 ± 19.5	264.65 ± 20.92	285.7 ± 22.2

Mean diff.	+0.1	−2.0	−3.70	−5.2	−3.4	−3.4	−5.0	−1.8	−4.7	−4.0	−5.0

*P* value	0.343	0.466	0.491	0.122	0.352	0.399	0.198	0.485	0.262	0.317	0.320

The values given in this table showed no statistically significant differences between the KC group and the control subjects (diff.: difference, cube vol.: cube volume, IS: inner superior, II: inner inferior, IT: inner temporal, IN: inner nasal, OS: outer superior, OI: outer inferior, OT: outer temporal, and ON: outer nasal).
